# Patient Care Technology Disruptions Associated With the CrowdStrike Outage

**DOI:** 10.1001/jamanetworkopen.2025.30226

**Published:** 2025-07-19

**Authors:** Jeffrey L. Tully, Sumanth Rao, Isabel Straw, Rodney A. Gabriel, Christopher A. Longhurst, Stefan Savage, Geoffrey M. Voelker, Christian J. Dameff

**Affiliations:** 1 Center for Healthcare Cybersecurity, University of California, San Diego, La Jolla; 2 Department of Anesthesiology, University of California, San Diego, La Jolla; 3 Department of Computer Science and Engineering, University of California, San Diego, La Jolla; 4 Department of Emergency Medicine, University of California, San Diego, La Jolla; 5 Department of Biomedical Informatics, University of California, San Diego Health, San Diego

## Abstract

**Importance:**

Modern health care depends on digital infrastructure. Widespread technology outages affecting health care delivery organizations may impact clinical care.

**Objective:**

To determine the availability of health care delivery organization internet-connected networks before, during, and after the faulty CrowdStrike software update of July 19, 2024.

**Design, Setting, and Participants:**

This cross-sectional study measured availability of health care delivery organization networks by performing scans on internet address ranges and Fast Healthcare Interoperability Resources (FHIR) end points corresponding to individual hospitals, spanning various sizes, geographic range, and organizational types, assessing responsivity to scans 2 weeks before, during, and 2 weeks after the CrowdStrike update. Hospitals affiliated with US-based health care delivery organizations that used the Epic electronic health record and that had publicly observable internet services, including FHIR end points, were included. Data were analyzed from July 19, 2024, to May 17, 2025.

**Exposure:**

A widely distributed faulty software update for enterprise cybersecurity software distributed on July 19, 2024, which, when installed on computers, resulted in a system crash.

**Main Outcomes and Measures:**

The primary outcome was loss of response to internet scanning techniques indicating computer system downtime. A secondary analysis was performed on unresponsive domains attempting to identify the function of the network services provided.

**Results:**

Among 2232 hospitals with available data, loss of response to internet scanning techniques immediately following the CrowdStrike update was observed in 759 hospitals (34.0%). A total of 1098 distinct network services with outages were identified, of which 631 (57.5%) were unable to be classified, 239 (21.8%) were direct patient-facing services, 169 (15.4%) were operationally relevant services, and 58 (5.3%) were research-related services.

**Conclusions and Relevance:**

This cross-sectional study of US hospitals found that a widespread technology outage was associated with outages in patient-facing network services integral to care delivery. These findings suggest that internet measurement techniques may be useful for surveillance and study of critical digital health care infrastructure.

## Key Points

**Question:**

What patient care outcomes were associated with a major international technology outage?

**Findings:**

In this cross-sectional study of 2232 hospitals with available data, network disruptions coinciding with a faulty cyber security software update on July 19, 2024, were measured at 759 US hospitals. Of the nearly 1100 internet-based services examined, 239 (21.8%) were characterized as corresponding with direct patient care functionality.

**Meaning:**

These findings suggest that widespread technology failures affecting health care infrastructure may have commensurate negative impacts on patient care systems.

## Teaser

This cross-sectional study describes the availability of health care delivery organization internet-connected networks before, during, and after a faulty cyber security software update on July 19, 2024.

## Introduction

Modern health care delivery depends on an increasingly complex digital infrastructure. The physician using an electronic health record (EHR), ordering and reviewing laboratory and radiographic studies, or capturing charges for subsequent reimbursement likely relies on a network of computers running software, connected to the wider internet, to care for patients.

This shift has brought improvements in the quality and efficiency of care but has also created a new source of risk.^1,2,3,4^ Examples of technology failures, both inadvertent and intentional, resulting in the disruption of patient care have proliferated in recent years.^5,6,7,8,9^ Independent of etiology, loss of critical systems often necessitates the use of downtime procedures, which are frequently underprepared for and are associated with increased likelihood of medical error.^10,11,12^ While the initial impact of an innocuous event, like a power failure, may result in the same need for downtime procedures as a targeted cybersecurity incident, the ultimate duration and scale of the disruptions may vary widely.^13^

Although cybersecurity incidents are an increasingly prominent source of technology failures, nonmalicious events, including unplanned network outages, failures in key infrastructure services, and software failures, can be equally disruptive. In particular, automated software updates, while critical to improving the function or security of systems, can result in widespread crashes if the update itself is faulty. Such outages have been documented with updates to EHR software, as well as with nonclinical software widely deployed in enterprises across many sectors.^14,15,16,17^

One such outage was recently associated with CrowdStrike, a cybersecurity software company, whose Falcon software is designed to monitor and protect computers of large commercial enterprises from cybersecurity threats. On July 19, 2024, a faulty update for Falcon was simultaneously distributed via the internet to millions of personal computers and servers running certain versions of the Windows operating system (Microsoft) and the Falcon software.

The update contained a programming error that caused computers that installed the update to reboot and crash. Installing the repair or patch for this update required direct manual access to each affected computer and could not be accomplished remotely through the internet—a time-intensive and laborious process resulting in downtimes of hours or even days for many organizations.^18,19^

The impact from the Falcon update outage was immediate and global.^20^ Disruptions were sustained across dozens of industries in dozens of countries: air travel saw the cancellation of thousands of flights worldwide, large banks and federal government systems in the US were impacted, and some factories and ports briefly shut down.^21^ Few sectors were spared, and health care was no exception, with large academic centers reporting an inability to access EHRs and the cancellation of elective surgeries.^22^ Emergency response services across multiple countries were adversely affected, and several of the largest laboratory vendors in the US had delays or were unable to process results.^23,24^

Today there is no public health surveillance system that monitors the well-being of critical health care technology systems. However, because such systems are part of a larger global internet infrastructure, their failures are frequently reflected in symptoms visible via the public internet. Indeed, a large body of existing research in applied computer science has developed techniques to infer and characterize various kinds of system failures based entirely on the results of diligent and focused internet measurements.^25,26,27,28^ Variants of these same techniques, when applied and specialized to hospital infrastructure, show promise in monitoring and quantifying disruptions to critical digital health care technology. We report the development of a system to monitor and detect disruptions in critical digital health care infrastructure.

## Methods

This cross-sectional study was deemed exempt from institutional review board review and informed consent by the University of California, San Diego, as this study had no human participants. This study is reported following the Strengthening the Reporting of Observational Studies in Epidemiology (STROBE) reporting guideline.^29^

### Study Design

This cross-sectional study was conducted between July 5, 2024, and August 3, 2024. Data for 2 weeks before (July 5-18), during (July 19), and after (July 20 to August 3) the CrowdStrike outage were collected and analyzed. US health care delivery organizations (HDOs) running Epic EHR software (Epic Systems) that had at least 1 publicly available Fast Healthcare Interoperability Resources (FHIR; The HL7 FHIR Foundation) internet end point were included.

### Data Sources

Data for this study were collected as part of a preexisting initiative to prospectively monitor hospital ransomware attacks with funding from the Advanced Research Projects Agency for Health. We collected and cataloged internet address ranges for HDOs and hospitals that use Epic as their EHR provider and that host external and public-facing internet services. We used a provider of historical internet measurement data (Censys), to match hospital domains to Internet Protocol (IP) ranges, filtering using Domain Name System and x.509 certificate information provided by Censys’s daily snapshot of the internet address space. Services running on the identified hosts were similarly enumerated. FHIR end points were identified from public data, including the Lantern Project,^30^ a publicly available resource from MITRE.

### Data Collection

Hospital IP range scans were performed using an open-source tool designed to probe open network ports of internet-connected hosts (ZMap version 4.2.0; University of Michigan).^31^ Address ranges were probed in rounds of 3 hours, and a positive scan (on any port) indicated an online host end point. Scans of FHIR end points were performed in rounds of 2.5 hours using a Python (Python Software Foundation) hypertext transfer protocol client. Positive scan results, ie, end point was operational and communicating with outside internet traffic, as well as negative scans, ie, system downtime, were recorded and stored on a secure internal server.

### Downtime Classification

Downtime for the address range scans was defined as the period between when a deviation from the normative count of positive IP scans (by a factor ≥2 SD) occurred to when it recovered. Downtime for a hospital or HDO was defined as the maximum downtime for any network on which it hosted any service.

Downtime for the FHIR end point scans was defined as the time between a negative scan and the return of a positive scan. End points in our list that had no positive scan the entire duration of the study were excluded. Downtime instances were then collated into a dataset for subsequent analysis of potential clinical effects.

### Clinical Translation

Once unresponsive network services were identified, further steps were taken to provide a clinical interpretation of the services related to each HDO or hospital to infer potential impacts on clinical operations. Four researchers independently analyzed 1098 affected services in the CrowdStrike outage dataset. The clinical relevance of the 1098 affected services was investigated through (1) directly visiting site Uniform Resource Locators (URLs), (2) Google dorking, a technique harnessing search engines to identify specific text in website code,^32^ and (3) Domain Name Service evaluation through terminal queries.^33^ Where further confirmation was needed, Client for URL requests were used to check site availability, hypertext transfer protocol status codes, and headers to attempt to determine the clinical function of an IT service. Viewing the page source provided additional insights, such as metadata with information on the services behind a login portal (eg, remote staff access portals for patient records) and hidden redirects.

Through the combination of these methods, each affected service was manually assigned a label detailing its function within clinical operations (eg, patient portal for health care record). Examples of each method and successfully identified results were collected and are provided in the eTable in Supplement 1. Based on the manual labels, each service was assigned 1 of 4 categories capturing its relevance to patient care: (1) patient facing (eg, radiological imaging systems), (2) operationally relevant (eg, staff scheduling systems), (3) research relevant (eg, research databases for clinical trial operations) and (4) not relevant or unknown (eg, donation pages for academic institutions). For categories 1 to 3, the service had to affect patient experience; thus, services such as research laboratory information webpages were excluded and placed in category 4. Services that could not be identified due to internal network or security restrictions or that were decommissioned and unavailable were also placed in category 4.

### Statistical Analysis

A deviation-from-baseline was established using a trailing 2-week window of 112 scans (8 hours per day × 14 days) of unique IP counts per hospital. Downtime events were flagged during the study period when the deviation met or exceeded 2 SD. This approach allowed consistent thresholds across institutions. For FHIR end points, we performed 2 comparisons using *t* tests. A 1-sample *t* test was used to determine whether the event count on the incident day represented a statistically significant outlier. A Welch 2-sample *t* test was used to compare the mean daily event counts between the preincident and postincident groups to account for potentially unequal variances between groups. A 2-sided *P* < .05 was considered statistically significant. As this is an observational study, corrections for multiple comparisons were not applied; instead, emphasis on clinical or infrastructural effects of disruptions was used in interpreting significance. Data were analyzed using Python version 3.12.8 (Python Software Foundation) from July 19, 2024, to May 17, 2025.

## Results

Immediately following the CrowdStrike update on July 19, 2024, a total of 759 of 2232 hospitals with available data (34.0%) experienced service disruptions detectable with our methods. These disruptions were identified solely by IP address space scans in 460 hospitals (60.6%), solely by FHIR end point scans in 206 hospitals (27.1%), and by both methods in 93 hospitals (12.3%).

**Figure 1.**
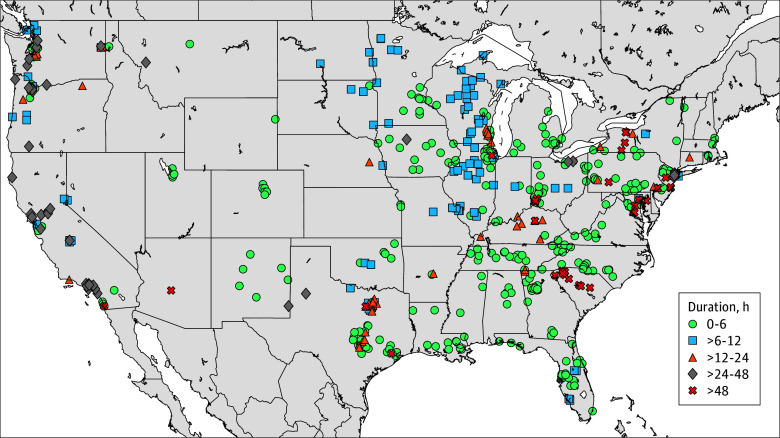
Geospatial Map of the Identified Health Care Delivery Organizations Inferred to Have Service Disruptions Using Address Space Scans Data points are color coded by the time to recovery following the CrowdStrike incident.

Further examination of affected domains across the hospitals yielded a total of 1098 individual disrupted digital services that were investigated for clinical relevance (eTable in Supplement 1). Figure 1 provides a more granular illustration of this downtime duration across the country. Most hospital services recovered within 6 hours (321 services [58.1%]), with a smaller number of hospitals (43 services [7.8%]) experiencing outages of more than 48 hours (Figure 1).

**Figure 2.**
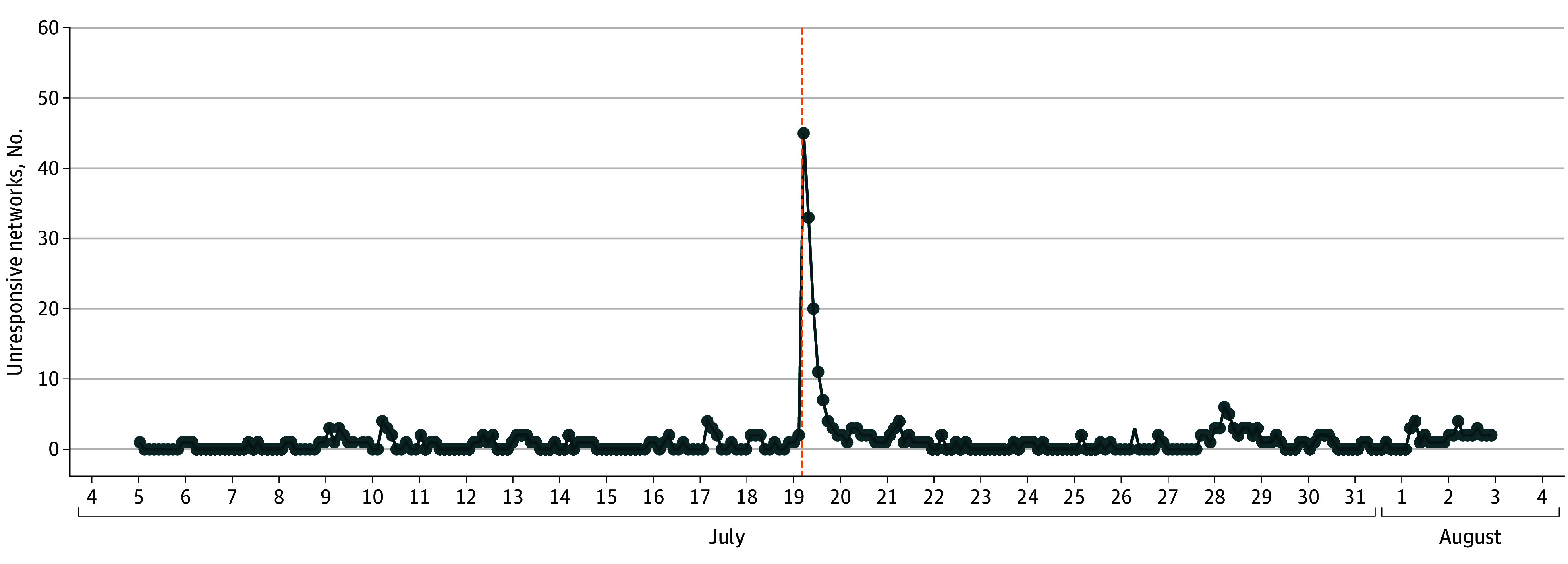
Unresponsive Health Care Delivery Organization (HDO) Fast Healthcare Interoperability Resource End Points Prior, During, and After the CrowdStrike Outage The vertical orange line marks the time point at which the CrowdStrike outage occurred, July 19, 2024.

We also observed that a set of 52 unique HDOs (representing 299 individual hospitals) failed to respond to our FHIR scans immediately following the July 19 update. The change in FHIR outage detection occurring during the CrowdStrike event is presented in Figure 2, which displays unresponsive HDO Epic FHIR end points resulting from scans conducted 2 weeks before and 2 weeks after the CrowdStrike outage.

The mean (SD) daily FHIR end point downtime was significantly increased during the CrowdStrike incident compared with the pre-event and postevent periods, with 6.0 (3.7) events prior, 128 events during, and 10.5 (9.1) events after the CrowdStrike incident (*P* < .001). There was no significant difference in the means between the pre-event and postevent periods. The median (IQR) observed downtime for all HDOs during the event was 5.1 (2.5-8.1) hours, with most HDOs recovering within 5 hours (31 of 52 HDOs [59.6%]).

### Outages in Clinical Systems

Of 1098 service outages, 239 (21.8%) were found to be patient facing, 169 (15.4%) were operationally relevant, 58 (5.3%) were research relevant, and 631 (57.5%) were not relevant or unknown (Table). Patient-facing services spanned imaging platforms, prehospital medicine health record systems, patient transfer portals, access to secure documentation, and staff portals for viewing patient details (Table). In addition to staff portals, we saw outages in patient access platforms across diverse hospital systems; these platforms, when operating as usual, allow patients to schedule appointments, contact health care practitioners, access laboratory results, and refill prescriptions. The not relevant or unknown category encompassed testing and staging environments for software that were in predeployment phases, donation pages for institutions, information pages on educational services, and educational resources for students (eg, medical and nursing students) (Table). The eTable in Supplement 1 provides further examples of successfully identified clinical services from the CrowdStrike outage dataset, with extracts of informative sections of source code, headers, and webpage details.

**Table.** Evaluation of Services With Outages and Their Relevant Clinical Utility

	Category of service			
	Patient facing	Operationally relevant	Research relevant	Not relevant / Unknown
Proportion of total affected services, No. (%) (n = 1098)	239 (21.8)	169 (15.4)	58 (5.3)	631 (57.5)
Examples of identified services and relevance to patient care	Staff portals for viewing patient health records, access to platforms for viewing prehospital clinical information, fetal monitoring systems and device management for telemonitoring, secure document transfer for inter-hospital transfers, access to imaging systems for viewing patient scans	Staff scheduling systems for regular and on call shifts, bill payment systems for health care insurers, clinical workforce management and optimization systems, portal for ordering facilities services, networked printers in the clinical environment, digital systems for establishing patient wait times and patient flow in the hospital environment	Access to databases for clinical trial operations, information websites for research laboratories, patient enrollment systems for rare disease research programs, staff login for clinical research management systems, REDCap research environment for academic centers, login portal for researchers at academic health centers	Testing and staging environments (preproduction), webpage for medical school alumni programs, donation websites for academic centers, medical education websites for students

## Discussion

In this cross-sectional study, we present a method of proactively monitoring digital signals corresponding to HDOs using well-established internet measurement techniques and report associated downtimes occurring at IP addresses corresponding to 759 hospitals during the CrowdStrike outage of 2024. To our knowledge, our data provide the first quantifiable insight into specific outcomes associated with this event. Our system measured disruptions in nearly 1100 unique services belonging to HDOs across the country. While most services (631 services [58.5%]) were either associated with nonclinical elements or unable to be categorized, characterization of the remaining elements allows for the most granular description of health care infrastructure outcomes associated with the CrowdStrike outage to our knowledge.

As digital systems are increasingly important element of health care infrastructure, technology failures can disrupt the delivery of care and increase risk to patient safety.^34,35,36,37,38,39,40^ Technology failures that affect a widely used system, such as the CrowdStrike Falcon program, or a cyberattack on a company serving a large market share of HDOs, like the ransomware attack on Change Healthcare, can cause disruption on a national scale.^41^ Information on the extent of impact, the locations of HDOs affected, and the types of patients most at risk when technology fails is often lacking or parceled out anecdotally in media reports. To our knowledge, no federal, regional, state, commercial, or trade association health care stakeholder or entity possesses the capability to assess in near–real time digital signals corresponding to the availability of national health care infrastructure technology.

A vast number of nonclinical systems are needed to sustain hospital operations, and outages were observed in critical logistics, human resources, and physical infrastructure during the CrowdStrike outage. Email applications, online staff schedules, productivity and project management tools, security cameras, payroll systems, physician credentialing software, remote access tools, virtual private networks, and cybersecurity controls were among the operationally relevant system outages observed, representing 15% of our dataset. Loss of these systems may indirectly affect patient care by exacerbating staffing shortages, degrading communication, preventing remote work, or increasing physical or cybersecurity vulnerabilities, and the disabled documentation and charge capture systems measured likely resulted in parallel challenges with clinical billing.

Among services with outages, 5% corresponded to clinical research enterprises. Specific tools, like REDCap (Vanderbilt) software used to create research databases, were inaccessible at some hospitals, and services belonging to identifiable, specific research laboratories studying immunology, oncology, epigenetics, environmental toxicology, and microbiomics experienced outages. Most concerning, clinical trial websites directly serving patients, as well as public health and regional registries used for tracking trauma, stroke, cardiac, and burn patients, suffered downtime.

Finally, we characterized 239 services (21.8%) whose outages may have had direct patient safety implications. A substantial number of hospitals lost important services involving their EHR, likely preventing physicians from accessing critical patient information, using automated ordering or clinical decision support, or easily viewing laboratory or radiology results. Similarly, externally facing patient health portals were disrupted, threatening to deprive patients access to their medical records, including new results or diagnoses, obstructing the ability to easily communicate with their clinicians or to be reminded about upcoming appointments and potentially even delaying care by obfuscating critical paperwork or authorizations. Services belonging to websites used by hospitals to provide patient education, such as websites containing important information and instructions for expectant mothers, appointment help for parents seeking neuropsychological testing for their children, and occupational health resources for employees, were also found to be down.

Foundational critical software platforms, including Picture Archiving and Communications Systems and Laboratory Information System, were disrupted at some hospitals, potentially impacting critical clinical workflows dependent on the rapid results of imaging and laboratory studies. A number of patient monitoring systems, including fetal monitors, cardiac telemetry systems, and behavioral health applications, experienced outages, which may have had regional effects on some clinical applications, like prehospital reporting and telemedicine systems used to deliver care across distance, during the disruptions.

Public health surveillance systems have been long established as effective and critical for the monitoring of acute and long-term epidemiologic phenomena, from infectious disease outbreaks to toxic exposures to maternal and child health outcomes, and have been implemented on scales ranging from a single community to global views.^42,43,44,45,46,47^ Similarly, the disciplines of disaster medicine and emergency incident response are predicated on the ability to collect data during and after disasters to analyze impact and develop future preparedness plans.^48,49,50,51^ Visibility and insight into the normal and abnormal state of digital health infrastructure is thus a requirement in seeking to understand the increasingly complex relationships between technology and the delivery of patient care and to meaningfully characterize the effects of technology failure. As approaches to monitor digital health infrastructures evolve, the information they produce may become useful for stakeholders charged with preparing for and responding to public health or infrastructure-related emergencies, helping to identify geographic regions, patient populations, or classes of services most likely to be impacted during a technology failure, allowing for targeted intervention designed to minimize harm.

### Limitations

This study has some limitations, and additional studies to develop these techniques are needed. Our method does not represent the definitive approach to obtaining comprehensive visibility of digital health care infrastructure, and limitations of this study include both classic problems in empirical internet measurement as well as challenges unique to health care delivery. The question of ground truth is inherent to a field where elements being measured are surrogates for the systems of interest. While downtime of an IP address corresponding to an EHR application programming interface strongly suggests disruption in the ability to use that EHR, without confirmation from clinicians at that institution, it is impossible to preclude a situation in which internal mitigations somehow enabled persistence of the underlying system. Similarly, while evidence exists to demonstrate that unplanned loss of clinical systems resulting in the transition to downtime procedures is associated with increase risk of error and decreased efficiency, generalizable clinical outcome data associated with such downtimes is correspondingly lacking. Technical considerations and limitations additionally give rise to seemingly arbitrary elements, such as the 2.5-hour scanning interval of the system and the fact that data are largely derived from health systems running the Epic EHR. Cloud-hosted digital services and those running behind firewalls are more challenging to measure with the approaches described; therefore, HDOs with less technically sophisticated networks and technology stacks may be overrepresented in these data. The development of further methods to allow for increased visibility into cloud dependencies and less publicly facing services is an area for future work. Large language models and other artificial intelligence tools may assist in identifying services and their associated functions at scale and with potentially increased accuracy.

## Conclusions

This cross-sectional study found an association between the 2024 CrowdStrike outage and many geographically diverse HDOs experiencing significant technical system downtime. Hospital recovery from the downtime was temporally varied. Prospective internet availability scanning of critical digital health care may serve as an early warning signal for adverse events, such as ransomware attack, data center failure, or faulty software, and could serve an important public health function as health care continues expanding its dependence on digital technology.

## Notes

Supplement 1. **eTable.** Example Table of Services Affected by CrowdStrike Outage and the Techniques Used to Translate the Services to Their Clinical Context

Supplement 2.
Data Sharing Statement
