# Errors in Results

**DOI:** 10.1001/jamanetworkopen.2025.31871

**Published:** 2025-08-13

In the Original Investigation titled “Patient Care Technology Disruptions Associated With the CrowdStrike Outage,”^1^ published July 19, 2025, there were errors in the restored services percentages in the Results. These should have been reported as 321 services (58.1%) restored within 6 hours and 43 services (7.8%) experiencing outages of more than 48 hours. This article has been corrected.^1^
