# Supplementary material for: Patient Care Technology Disruptions Associated With the CrowdStrike Outage
Source: JAMA Netw Open. 2025 Jul 19;8(7):e2530226. doi: 10.1001/jamanetworkopen.2025.30226 (PMC12276631; doi:10.1001/jamanetworkopen.2025.30226)
Supplement: Supplement 1. — eTable. Example Table of Services Affected by CrowdStrike Outage and the Techniques Used to Translate the Services to Their Clinical Context [file jamanetwopen-e2530226-s001.pdf]

Supplemental Online Content

Tully JL, Rao S, Straw I, et al. Patient care technology disruptions associated with the CrowdStrike outage. *JAMA Netw Open*. 2025;8(7):e2530226. doi:10.1001/jamanetworkopen.2025.30226

eTable. Example Table of Services Affected by CrowdStrike Outage and the Techniques Used to Translate the Services to Their Clinical Context

This supplemental material has been provided by the authors to give readers additional information about their work.

© 2025 Tully JL et al. *JAMA Network Open*.

eTable1: Example table of services affected by Crowdstrike Outage and the techniques used to translate the services to their clinical context. Column 1 details the service from the Crowdstrike Outage Dataset that was investigated, and the category that was assigned following investigation (e.g. patient facing). Column 2 provides the technique for obtaining information on the service and the relevant obtained information and the final column provides the clinical translation of this service in the context of patient care. Identifiable information has been removed and replaced with "XX" to protect the identity of individual organizations.

Service & Categorization	Technique used for Clinical Translation & Extracted Information	Clinical Translation and Key Information Derived
**Patient Facing:**<br>urimages.**XX**.org	**Technique:** *View page source*<br><br>**Extracted Information:**<br>*<title>Merge Universal Viewer</title>*<br>*<script src="Scripts/LaunchExamImporter.js?ver=8.4.1.34" type="text/javascript"></script>*<br>*<input id="downloadPacsGatewayButton" type="button" value="Go"*<br>*onclick="onGetPacsGateway(); return false;" class="imageSkinnedButton_Normal nonRegisterUserButton" />*	The service leads to a login page for PACS (Picture Archiving and Communication System), which is used for storing and retrieving digital medical images. Thus, the service relates to patient radiological imaging services at XX health system, and loss of the service would impact clinical care decisions dependent on imaging facilities at the hospital (e.g. management of acute surgical conditions).
**Patient Facing:**<br>chi.**XX**.net	**Technique:** *Terminal CURL requests [curl -L chi.XX.net]*<br><br>**Extracted Information:**<br>*<meta name="description" content="Take your health with you: Schedule appointments, E-mail your doctor, Get lab results, Track your health history, Request prescription refills, Pay your bills online and much more"/>*<br>*Patient portal for lab results and contacting doctor*	The extracted code illustrates that the login page leads to a patient portal where patients can schedule appointments, get lab test results, refill prescriptions and track health appointments.
**Operationally Relevant:**<br>XX.careforceconnection.org	**Technique:** *Google Dork - site:XX.org "Careforce Connection"*<br><br>**Extracted Information:**<br>*Search Results:*<br>*Employee Resources XX Healthcare of XX https://www.XX.org › staff*	The search results demonstrate a system for scheduling staff shifts and providing employee resources, impacting the operations of the hospital and the allocation of practitioners to clinical shifts.

© 2025 Tully JL et al. *JAMA Network Open.*

	From accessing the Remote Portal and Webmail to Careforce Connection, there's an abundance of resources for Children's employees.<br><br>*Staff Scheduling and Employee Resources for XX Healthcare of XX*	
**Patient Facing:** data.XXtrauma.org	**Technique:** *Visited site directly*<br><br>**Results on Site:** "Patient Registry tools seamlessly integrate pre-hospital EMS Incidents to the hospital and registry, ensuring patient centric data aggregation"	Integrates medical data from ambulances to hospitals, providing information on pre hospital events for trauma and emergency patients.
**Patient Facing:** https://e.XX.org/mychart/Authentication/Login?	**Technique:** *Visited site directly*<br><br>**Results on Site:** "Patient Registry tools seamlessly integrate pre-hospital EMS Incidents to the hospital and registry, ensuring patient centric data aggregation"	Patient portal for access lab reports, **request prescription refills,** manage appointments and contact doctor
**Patient Facing:** elite.XX.net	**Technique:** *Terminal Curl Request - curl -L https://XX.XX.org*<br><br>**Result:**<br>Gave result of ImageTrend Elite service, sensitive details removed to protect identity of organization.	Imaging System: electronic patient care reporting (ePCR) and records management system used by EMS and fire departments to document emergency medical responses and share details with hospitals.
**Patient Facing:** ema.XX.org	**Technique:** *Terminal Curl Request*<br><br>**Result:**<br>*Relevant elements of output:*<br>*<title>Intel® Endpoint Management Assistant</title>*<br>*Javascript: window.IntelEMA.ApiUrl = 'https://ema.XXX.org/api/latest/*<br>*Remote management scripts indicate terminal control:*<br>*<script type="text/javascript" src="/ema_terminal.js"></script>*	EMA is used for **IT device management**, ensuring efficient management of **hospital endpoints** (PCs, servers, medical devices). Thus, an outage may result in IT staff may be unable to remotely monitor, troubleshoot, or manage hospital computers and medical device endpoints.
**Patient Facing:** XX-hh.XX.gov	**Technique:** *Terminal Curl Request*<br>*curl -L XX-XX.XX.gov*	Allows hospitals receiving ambulances to receive access to the electronic patient care records for transferred

© 2025 Tully JL et al. *JAMA Network Open*.

	**Result:** Relevant output:<br>*<title>XX Hospital HUB</title>*<br>*<div><p>The XX State Ambulance Reporting system (XX) Hospital Hub allows all receiving facilities access to the electronic patient care records for all EMS transports in the State of XX transported to their facility.*	patients. Thus, this resource is important for patient transfers.
**Patient Facing**<br>prod.XX.org	**Technique:** *View page source*<br><br>**Key results from page source:**<br><li xmlns="">A-1 How to Use This Manual <br><li xmlns="">A-2 XX and XX XX History <li xmlns="">A-3 The Behavioral Health Aide Program	Page source reveals that through LoginPage is the The Behavioral Health Aide Program for the XX patient population. Providing links to Referrals, Consent to Treatment, Mandatory Reporting and Screening for different conditions.

© 2025 Tully JL et al. *JAMA Network Open*.
