# Supplementary material for: Patient Care Technology Disruptions Associated With the CrowdStrike Outage
Source: JAMA Netw Open. 2025 Jul 19;8(7):e2530226. doi: 10.1001/jamanetworkopen.2025.30226 (PMC12276631; doi:10.1001/jamanetworkopen.2025.30226)
Supplement: Supplement 2. — Data Sharing Statement [file jamanetwopen-e2530226-s002.pdf]

Data Sharing Statement

Tully. Patient Care Technology Disruptions Associated With the CrowdStrike Outage. *JAMA Netw Open*. Published July 19, 2025. doi:10.1001/jamanetworkopen.2025.30226

Data
Data available: Yes
Data types: Deidentified participant data
How to access data: Requests for access to deidentified data may be sent to jtully@health.ucsd.edu
When available: With publication

Supporting Documents
Document types: None

Additional Information
Who can access the data: Researchers whose proposed use of the data has been approved
Types of analyses: Any purpose
Mechanisms of data availability: With signed institutional data sharing agreement
